# MeCP2 facilitates breast cancer growth via promoting ubiquitination-mediated P53 degradation by inhibiting RPL5/RPL11 transcription

**DOI:** 10.1038/s41389-020-0239-7

**Published:** 2020-06-01

**Authors:** DongDong Tong, Jing Zhang, XiaoFei Wang, Qian Li, Li Ying Liu, Juan Yang, Bo Guo, Lei Ni, LingYu Zhao, Chen Huang

**Affiliations:** 1 Department of Cell Biology and Genetics/Key Laboratory of Environment and Genes Related to Diseases, School of Basic Medical Sciences, Xi’an Jiaotong University Health Science Center, Xi’an, Shaanxi 710061 China; 2 Institute of Genetics and Developmental Biology, Translational Medicine Institute, School of Basic Medical Sciences, Xi’an Jiaotong University Health Science Center, Xi’an, Shaanxi 710061 China; 3 Department of Clinical Medicine, Medical College of Yan’an University, Yan’an, Shanxi 716000 China

**Keywords:** Breast cancer, Epigenetics, Breast cancer, Epigenetics, Ubiquitylation

## Abstract

Methyl-CpG-binding protein 2 (MeCP2) facilitates the carcinogenesis and progression of several types of cancer. However, its role in breast cancer and the relevant molecular mechanism remain largely unclear. In this study, analysis of the Cancer Genome Atlas (TCGA) data that MeCP2 expression was significantly upregulated in breast cancer tissues, and high MeCP2 expression was correlated with poor overall survival. Knockdown of MeCP2 inhibited breast cancer cell proliferation and G1–S cell cycle transition and migration as well as induced cell apoptosis in vitro. Moreover, MeCP2 knockdown suppressed cancer cell growth in vivo. Investigation of the molecular mechanism showed that MeCP2 repressed RPL11 and RPL5 transcription by binding to their promoter regions. TCGA data revealed significantly lower RPL11 and RPL5 expression in breast cancer tissues; additionally, overexpression of RPL11/RPL5 significantly suppressed breast cancer cell proliferation and G1–S cell cycle transition and induced apoptosis in vitro. Furthermore, RPL11 and RPL5 suppressed ubiquitination-mediated P53 degradation through direct binding to MDM2. This study demonstrates that MeCP2 promotes breast cancer cell proliferation and inhibits apoptosis through suppressing RPL11 and RPL5 transcription by binding to their promoter regions.

## Introduction

Breast cancer is a major malignant tumor and the leading cause of cancer-related death among women worldwide^1,2^. Many patients may experience metastasis, with cancer cells spreading to the lungs, brain, liver, bone marrow, and lymph nodes^3^. Improvements in diagnostic accuracy and the development of antitumor drugs have dramatically decreased breast cancer mortality. Nevertheless, satisfactory therapeutic effects have yet to be achieved because it is an extremely complex disease. This complexity hampers the exploration of mechanisms underlying carcinogenesis and cancer progression, which are multistep processes involving many oncogenes and anti-oncogenes^4^. Some studies have shown that abnormal transcriptional activities of oncogenes and tumor suppressor genes are involved in breast cancer tumorigenesis^5^. Therefore, understanding the transcriptional regulation of cancer-related genes is crucial for breast cancer diagnosis and treatment.

Methyl-CpG-binding protein 2 (MeCP2), an important member of the methyl-CpG-binding domain (MBD) family, includes two main domains: an MBD and a transcriptional repression domain (TRD)^6^. MeCP2 is an X-linked gene whose mutation leads to multiple phenotypes that fall under the umbrella of Rett syndrome. As a crucial epigenetic regulator, MeCP2 regulates chromatin organization and gene transcription by binding to the methylated DNA sites of gene promoter regions^7–9^. It acts not only as a transcriptional repressor by selectively binding methylated CpG dinucleotides and recruiting co-repressors, such as histone deacetylases and Sin3A, but also as a transcriptional activator by selectively binding methylated CpG islands and recruiting activators, such as CREB1^10^. MeCP2 is reported as a frequently amplified oncogene in several cancer types, such as colorectal, lung, cervical, breast, and uterine cancers^11^. In a previous study, MeCP2 was upregulated in breast cancer and bound to hypermethylated tumor suppressors, which indicated that MeCP2 acted as an oncogene during breast cancer proliferation^12–15^. As revealed in our previous studies, MeCP2 facilitates gastric cancer cell proliferation and inhibits cell apoptosis through suppressing FOXF1/MYOD1 transcription and promoting GIT1 transcription by binding the methylated CpG islands of their promoter regions^16,17^. Given the existing studies, the role of MeCP2 in breast cancer has not been precisely examined. In particular, the molecular mechanism by which MeCP2 promotes tumor proliferation remains unclear.

In the present study, we investigated the role and molecular mechanism of MeCP2 in breast cancer proliferation. By analyzing the Cancer Genome Atlas (TCGA) data, we found that MeCP2 expression was significantly upregulated in breast cancer, and its expression level was correlated with the clinicopathological features. MeCP2 facilitated breast cancer cell proliferation and inhibited cell apoptosis through suppressing RPL11 and RPL5 expression by binding to their promoter regions, thereby promoting ubiquitination-mediated P53 degradation. Our findings suggest that MeCP2 may be a novel therapeutic target for breast cancer treatment.

## Results

### MeCP2 was upregulated in breast cancer and promoted cell proliferation and migration in vitro

**Fig. 1:**
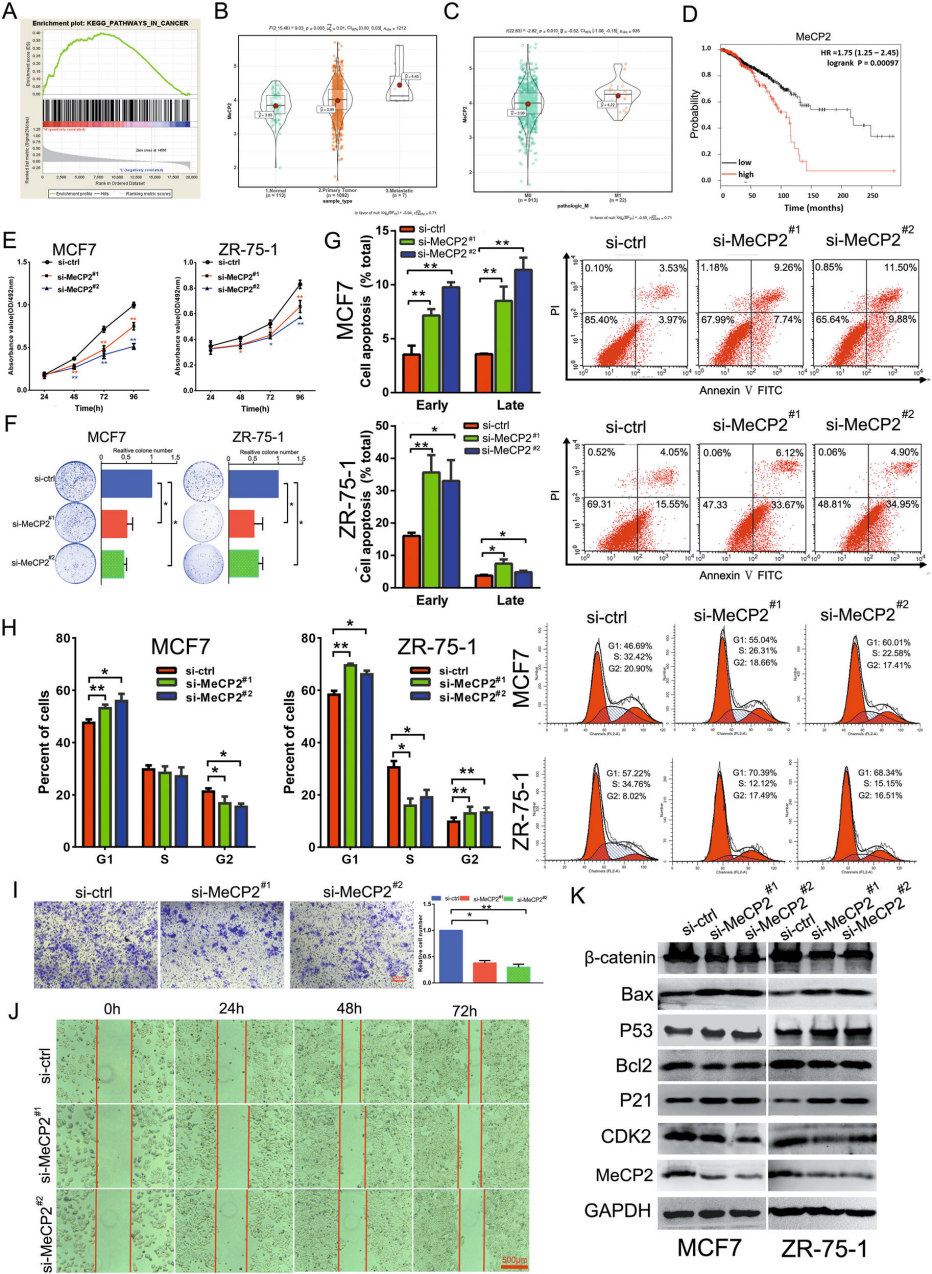
MeCP2 is upregulated in breast cancer tissues and promotes cancer proliferation. **a** GSEA of MeCP2 in breast cancer based on TCGA data. The results showed significant enrichment of the gene set involving the cancer-related signaling. **b** Bioinformatics analysis of MeCP2 expression in breast cancer and para-carcinoma tissues based on TCGA data. *P* < 0.01. **c** MeCP2 expression in M0 stage and M1 stage breast cancer based on TCGA data. *P* < 0.05. **d** Kaplan–Meier curves of breast cancer survival by MeCP2 expression based on TCGA data. **e** MTT assay of cell proliferation by at 24, 48, 72 and 96 h after transfection with MeCP2 siRNA. *P < 0.05, **P < 0.01. **f** Colony formation assay 14 days after transfection. *P < 0.05. **g** Cell apoptosis by flow cytometry, visualized using Annexin-V/PI staining. *P < 0.05, **P < 0.01. **h** Flow cytometry of cell cycle, visualized by PI staining. *P < 0.05, **P < 0.01. **i** Transwell assay of the effect of silencing MeCP2 on MCF7 cell migration. *P < 0.05, **P < 0.01. **j** Wound-healing assay of the effect of silencing MeCP2 on MCF7 cell migration. **k** Western blotting of the expressions of β-catenin, Bcl2, P53, Bax, CDK2, P21, and MeCP2 in MCF7 and ZR-75-1 cells after transfection with MeCP2 siRNAs.

To investigate the possible driving mechanism of breast cancer, we evaluated the MeCP2-related enrichment pathways by gene set enrichment analysis (GSEA) and found that the cancer-related pathway was significantly positively related to MeCP2 (Fig. 1a). Principal component analysis indicated that the expression of genes involved in this pathway differed between normal and breast cancer tissues (Supplementary Fig. S1A, B). TCGA data showed that MeCP2 expression was significantly higher in breast cancer tissues (*n* = 1099) than in normal breast tissues (*n* = 113) (Fig. 1b), and high MeCP2 expression was associated with M stage (Fig. 1c). Concordantly, statistical analysis showed that patients with higher MeCP2 expression had poorer overall survival (Fig. 1d). To further investigate the biological effect of MeCP2 on breast cancer in vitro, we used siRNAs to silence endogenous MeCP2 expression in breast cancer cell lines MCF7 and ZR-75-1. The qRT-PCR and western blotting results showed that MeCP2 siRNAs significantly downregulated MeCP2 expression at both mRNA and protein levels in these cells (Supplementary Fig. S2A, B and Fig. 1k). These results, along with cell viability and colony formation assays, revealed that silencing MeCP2 significantly inhibited breast cancer cell proliferation (Fig. 1e, f). MeCP2 siRNAs also increased early and late apoptotic cells (Fig. 1g) and induced G1 cell-cycle arrest (Fig. 1h). Furthermore, silencing MeCP2 remarkably suppressed MCF7 and ZR-75-1 cell migration (Fig. 1i, j). The levels of β-catenin, Bcl2, and CDK2 decreased in breast cancer cells treated with MeCP2 siRNAs compared to those with control siRNA, whereas the expression levels of Bax, P21, and P53 increased (Fig. 1k).

### MeCP2 inhibited RPL11 and RPL5 transcription by binding to their promoters

**Fig. 2:**
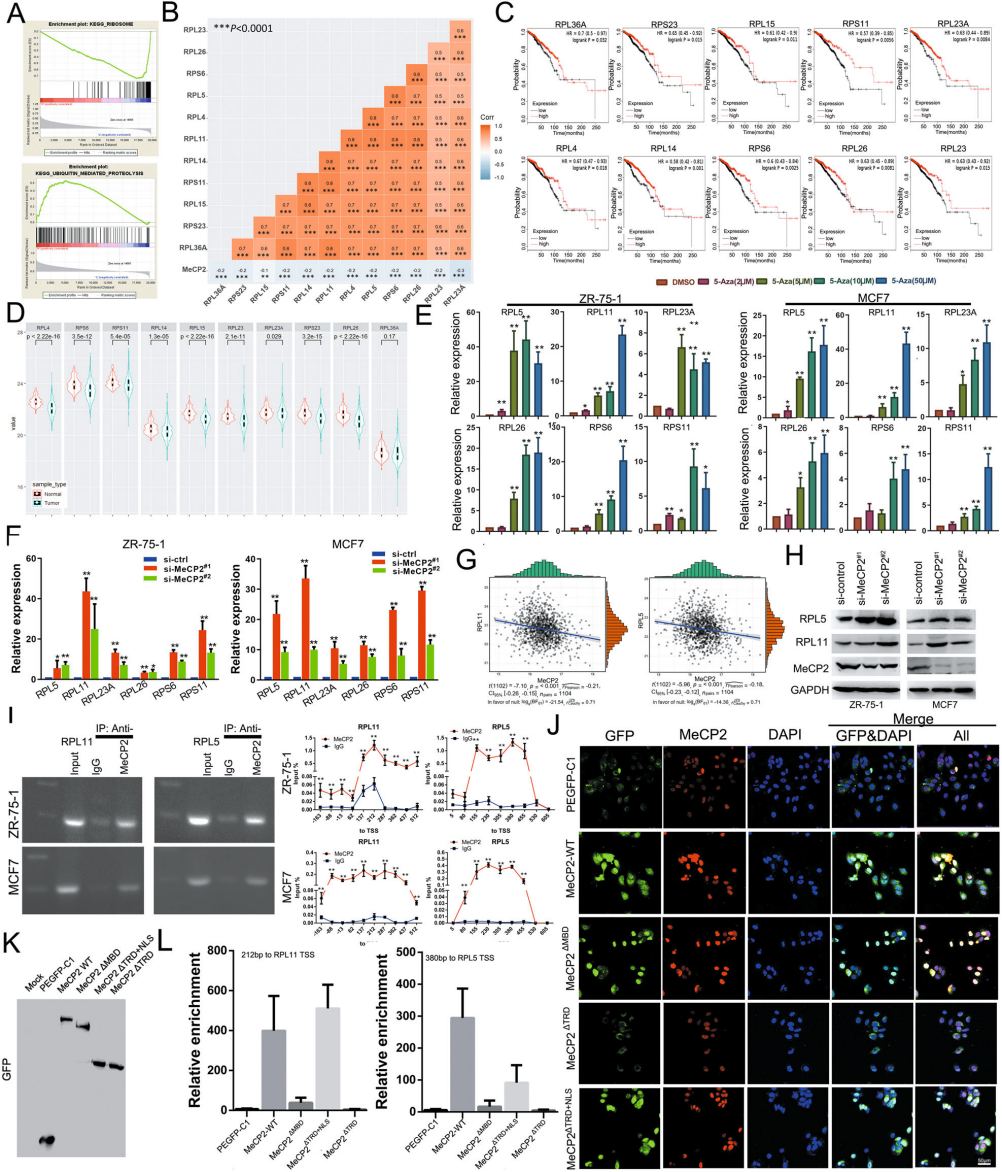
MeCP2 represses the expressions of RPL11 and RPL5 in breast cancer cells by binding to their promoter regions. **a** GSEA of MeCP2 in breast cancer based on TCGA data. The results showed significant enrichment of the gene set involving ribosome- and ubiquitin-mediated biological pathways. **b** Correlation between MeCP2 expression and the expressions of ribosomal proteins (RPs) in breast cancer. ***P < 0.001. **c** Kaplan–Meier curves of breast cancer survival by related RPs. **d** Analysis of RP expressions in breast cancer and para-carcinoma tissues. **e** mRNA expressions of RPL5, RPL11, RPL23A, RPL26, RPS6 and RPS11 in ZR-75-1 and MCF7 cells treated with 5-Aza or dimethyl sulfoxide (DMSO). *P < 0.05, **P < 0.01. **f** mRNA levels of RPL5, RPL11, RPL23A, RPL26, RPS6 and RPS11 in ZR-75-1 and MCF7 cells transfected with MeCP2 siRNAs. *P < 0.05, **P < 0.01. **g** Correlation between MeCP2 expression and RPL11 or RPL5 expression in breast cancer based on TCGA data. **h** The protein expressions of RPL11 and RPL5 in MCF7 and ZR-75-1 cells transfected with MeCP2 siRNAs. **i** ChIP PCR gel electrophoresis analysis (left) and ChIP RT-PCR (right) of RPL11 and RPL5 with anti-MeCP2 antibody in MCF7 and ZR-75-1 cells. **j** Immunofluorescence of the expression of plasmids MeCP2-WT, MeCP2ΔMBD, MeCP2ΔTRD and MeCP2ΔTRD+NLS in MCF7 cells. The nuclei were stained by DAPI. **k** Western blot of the expression of the plasmids in MCF7 cells. **l** ChIP-PCR assay for the target sequences of the promoters of RPL11 and RPL5, and simultaneous ChIP analysis of poly II A binding to GAPDH promoter as internal reference.

**Fig. 3:**
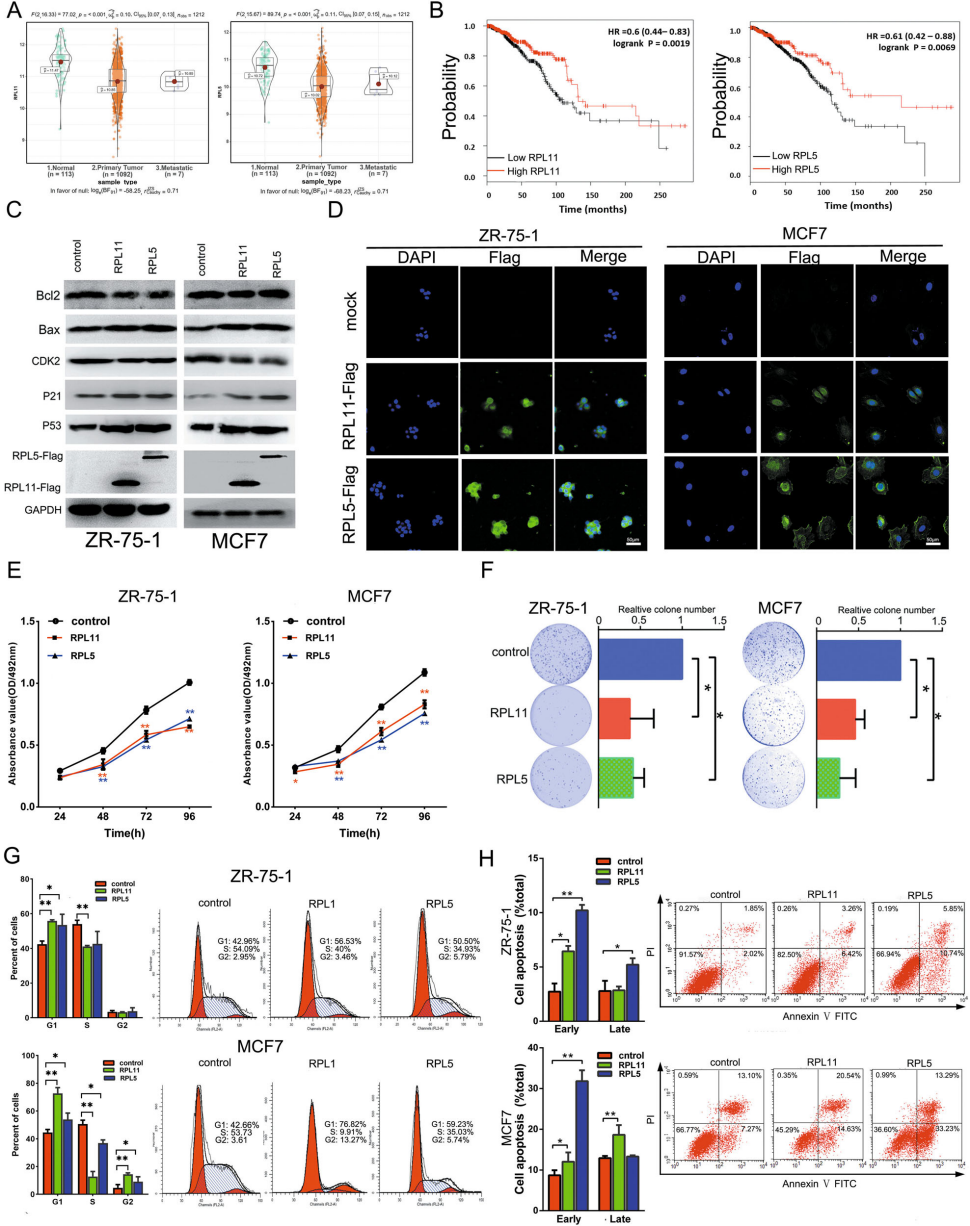
RPL11 and RPL5 inhibits breast cancer cell proliferation and induced apoptosis. **a** Bioinformatics analysis of RPL11 and RPL5 expressions in breast cancer and para-carcinoma tissues based on TCGA data. **b** Kaplan–Meier curves of breast cancer survival by RPL11 and RPL5 expressions based on TCGA data. **c** Expressions of Bcl2, P53, Bax, CDK2, P21, RPL11, and RPL5 in cells transfected with RPL11 or RPL5 overexpression vector. **d** Immunofluorescence of FLAG-tagged plasmids expressing RPL11 and RPL5 in MCF7 and ZR-75-1 cells. The nuclei were stained by DAPI. **e** MTT assay of cell proliferation at 24, 48 and 72, 96 h after transfection with RPL11 or RPL5 overexpression vector. *P < 0.05, **P < 0.01. **f** Colony formation assay 14 days after transfection. *P < 0.05. **g** Flow cytometry analysis of cell cycle in MCF7 and ZR-75-1 cells. *P < 0.05, **P < 0.01. **h** Flow cytometry analysis of cell apoptosis after transfection with RPL11 or RPL5 overexpression vector. *P < 0.05, **P < 0.01.

Enrichment pathway analysis showed that MeCP2 could significantly inhibit ribosome-mediated biological pathways and regulate ubiquitin-mediated pathways (Fig. 2a and Supplementary Fig. S3A, B). Additionally, our analysis of the specified ribosomal proteins (RPs), which were demonstrated in the BioGRID database as MDM2-P53 pathway mediators or as interacting with MDM2^18^, revealed that MeCP2 expression was correlated with RP expression, including RPL36A, RPS23, RPL15, RPS11, RPL23A, RPL4, RPL14, RPL11, RPL5, RPS6, RPL26, and RPL23 (Fig. 2b, g). TCGA data showed that high levels of these RPs were associated with high survival probability in breast cancer patients (Fig. 2c, 3b), and excepted RPL36A, their expression levels were suppressed (Fig. 2d, 3a). Analysis of six RPs that are major inhibitors of P53 ubiquitination, including RPL11, RPL5, RPL23A, RPS6, RPS11, and RPL26, showed that as the ratio of MeCP2 to RP increased, survival probability decreased (Supplementary Fig. S3C). To verify these findings, we treated MCF7 and ZR-75-1 cells with the methylation inhibitor 5-aza-2′-deoxycytidine (5-Aza) and found that 5-Aza at concentrations of 5, 10, 50μm dramatically upregulated the mRNA expression of the six RPs in the cells (Fig. 2e). Meanwhile, silencing MeCP2 remarkably increased the mRNA levels of these RPs (Fig. 2f).

We further examined the protein expression of RPL5 and RPL11 in MCF7 and ZR-75-1 cells and found that they were significantly upregulated after transfection with MeCP2 siRNAs (Fig. 2h). Chromatin immunoprecipitation (ChIP)-PCR assay revealed that MeCP2 bound to the promoters of RPL11/RPL5 in the cells (Fig. 2i). We constructed GFP-MeCP2 plasmids, including MeCP2-WT, MeCP2ΔMBD, MeCP2ΔTRD, and MeCP2ΔTRD+NLS, which were transfected into cancer cells. Immunofluorescence and western blotting assays confirmed successful plasmid expression in MCF7 cells (Supplementary Fig. S3D and Fig. 2j, k). ChIP-PCR assay revealed that MeCP2-WT and MeCP2ΔTRD+NLS could capture target sequences of the promoter regions of RPL11 and RPL5 (Fig. 2l and Supplementary Fig. S3E). TCGA data analysis indicated that the methylation levels of the CpG sites in the promoter region of either RPL11 or RPL5 were negatively correlated with their respective expression (Supplementary Fig. S3F). These results suggested that MeCP2 repressed RPL11 and RPL5 expression by binding to their promoters.

### RPL11 and RPL5 suppressed breast cancer cell growth and induced cell apoptosis

To further explore the biological effect of RPL11 and RPL5 in breast cancer, we analyzed their expression using TCGA data. It was found that both RPL11 and RPL5 expression levels were significantly lower in breast cancer tissues (*n* = 1099) than in normal breast tissues (*n* = 113) (Fig. 3a). Consistently, patients with higher RPL11 and RPL5 expression had longer overall survival (Fig. 3b).

We constructed RPL11- and RPL5-overexpressing plasmids, transfected them into MCF7 and ZR-75-1 cells, and confirmed plasmid expression of RPL11 and RPL5 in these cells by western blotting and Immunofluorescence assay (Fig. 3c, d). Furthermore, Results showed that Bcl2 and CDK2 levels decreased in the RPL11/RPL5-overexpressing breast cancer cells, whereas the expression of Bax, P21, and P53 increased in MCF7 and ZR-75-1 cells. Overexpression of RPL11/RPL5 significantly suppressed MCF7 and ZR-75-1 cell proliferation, as evidenced by both cell viability and colony formation assays (Fig. 3e, f). This overexpression also induced G1 cell-cycle arrest (Fig. 3g). Overexpression of RPL11 and RPL5 increased early apoptotic cells in both ZR-75-1 and MCF7 cells, RPL11 induced late apoptotic in MCF7 cells, and RPL5 facilitated late apoptotic in ZR-75-1 cells (Fig. 3h). These findings suggested that RPL11 and RPL5 could inhibit breast cancer cell proliferation and induce apoptosis.

### RPL5 and RPL11 delay P53 ubiquitination in breast cancer cells by binding MDM2

**Fig. 4:**
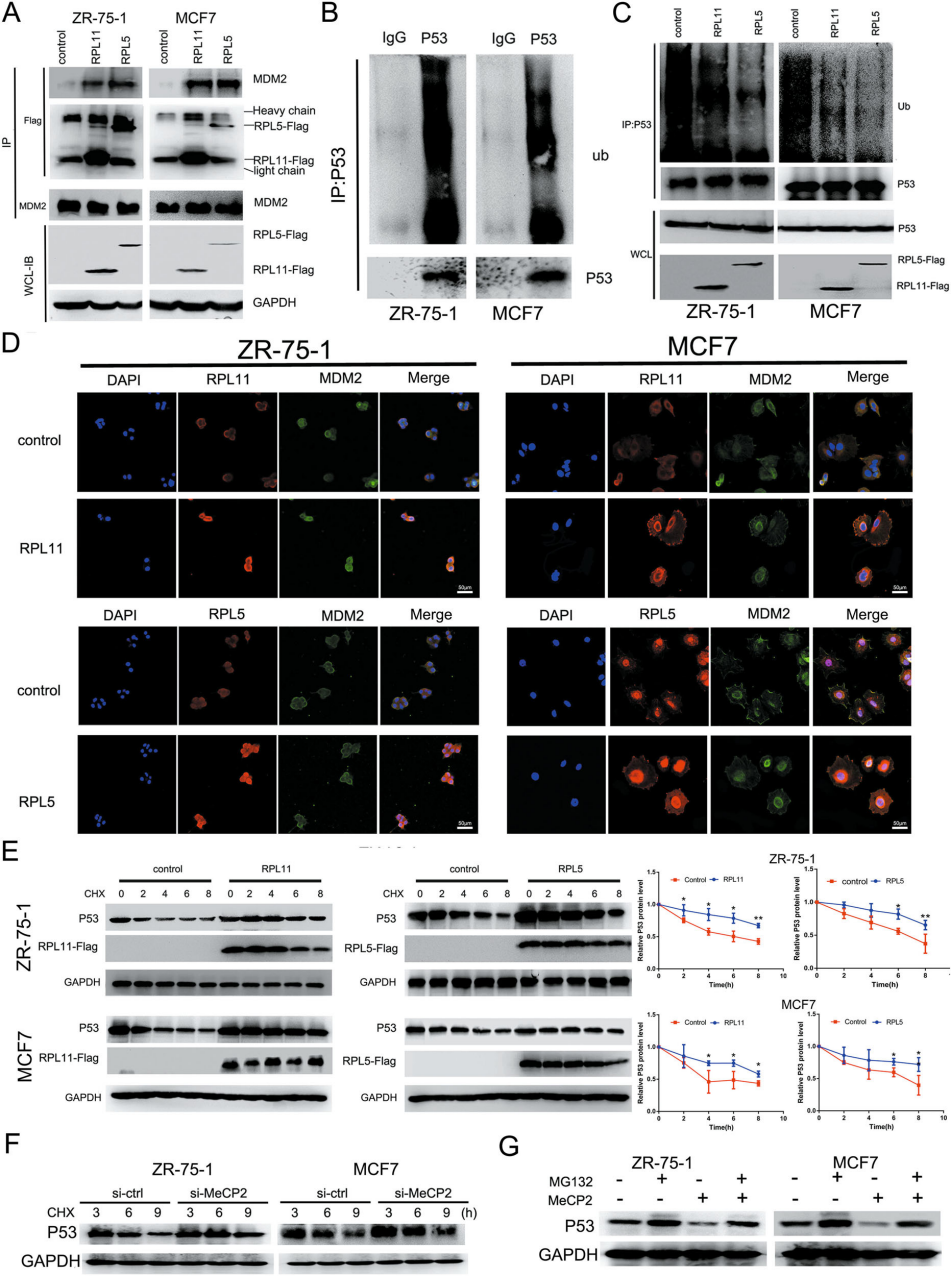
RPL11 and RPL5 suppress P53 degradation via binding to MDM2. **a** Co-immunoprecipitation of MDM2 binding to FLAG-tagged RPL11 or RPL5 in MCF7 and ZR-75-1 cells. Cells transfected with FLAG-tagged RPL11 or RPL5 were used in immunoprecipitation with MDM2 or FLAG antibodies, followed by immunoblot assays. **b** Anti-Ub immunoblotting of P53 ubiquitination in MCF7 and ZR-75-1 cells. **c** P53 ubiquitination in MCF7 and ZR-75-1 cells with or without RPL11/RPL5 transfection. **d** Confocal laser scanning microscopy for co-localization of MDM2/RPL11 or MDM2/RPL5 in MCF7 and ZR-75-1 cells transfected with RPL11 or RPL5 expression vector. **e** Half-life analysis of P53 protein in control and RPL5/RPL11 overexpressed MCF7 and ZR-75-1 cells. 10 μg/ml cycloheximide (CHX) was used to prevent de novo P53 biosynthesis. Left: gel analysis results; right: quantified results (P53 level was quantified and normalized to GAPDH expression). *P < 0.05, **P < 0.01. **f** Half-life analysis of P53 protein expression in control and MeCP2 siRNA transfected cells. **g** The P53 protein level in cells transfected with MeCP2 overexpression vector and proteasome inhibitor MG-132.

It has been reported that RPL5 and RPL11 act as P53 activators by partially abolishing the E3 ubiquitin ligase activity of MDM2 in some tumors. In this study, we aimed to determine whether RPL5 and RPL11 might regulate P53 activity by overcoming MDM2 inhibition in breast cancer. We tested whether RPL5/RPL11 could bind to MDM2 using a set of co-immunoprecipitation-immunoblot assays. Binding assay was also performed after transfecting MCF7 and ZR-75-1 cells with FLAG-tagged plasmids expressing RPL11 and RPL5. It was found that MDM2 was co-immunoprecipitated with RPL11 and RPL5 (Fig. 4a). P53 ubiquitination was observed in MCF7 and ZR-75-1 cells, which decreased after FLAG-tagged plasmid transfection (Fig. 4b, c). Confocal laser scanning microscopy confirmed the co-localization of RPL11/RPL5 and MDM2 in MCF7 and ZR-75-1 cells after transfection with the RPL11 and RPL5 overexpression vectors (Fig. 4d). To verify that RPL5 and RPL11 promote P53 stability, we treated the breast cancer cells with cycloheximide after transfection with the RPL11/RPL5 overexpression or control vectors. The results showed that the half-life of P53 in cells transfected with RPL11 or RPL5 overexpression vector was prolonged compared with control vector-transfected cells, indicating that RPL11 and RPL5 could inhibit P53 degradation (Fig. 4e). Moreover, P53 protein expression in cells transfected with MeCP2 siRNAs was higher than in those with control siRNA (Fig. 4f). We also treated cancer cells with proteasome inhibitor (MG-132) after transfection with MeCP2 overexpression vector and found that P53 protein expression was lower in such cells than in those transfected with the control vector (Fig. 4g). These results indicated that RPL11 and RPL5 could delay ubiquitination-mediated P53 degradation by directly binding to MDM2.

### MeCP2 facilitated breast cancer cell growth via inducing P53 degradation by inhibiting RPL11/RPL5 expression

**Fig. 5:**
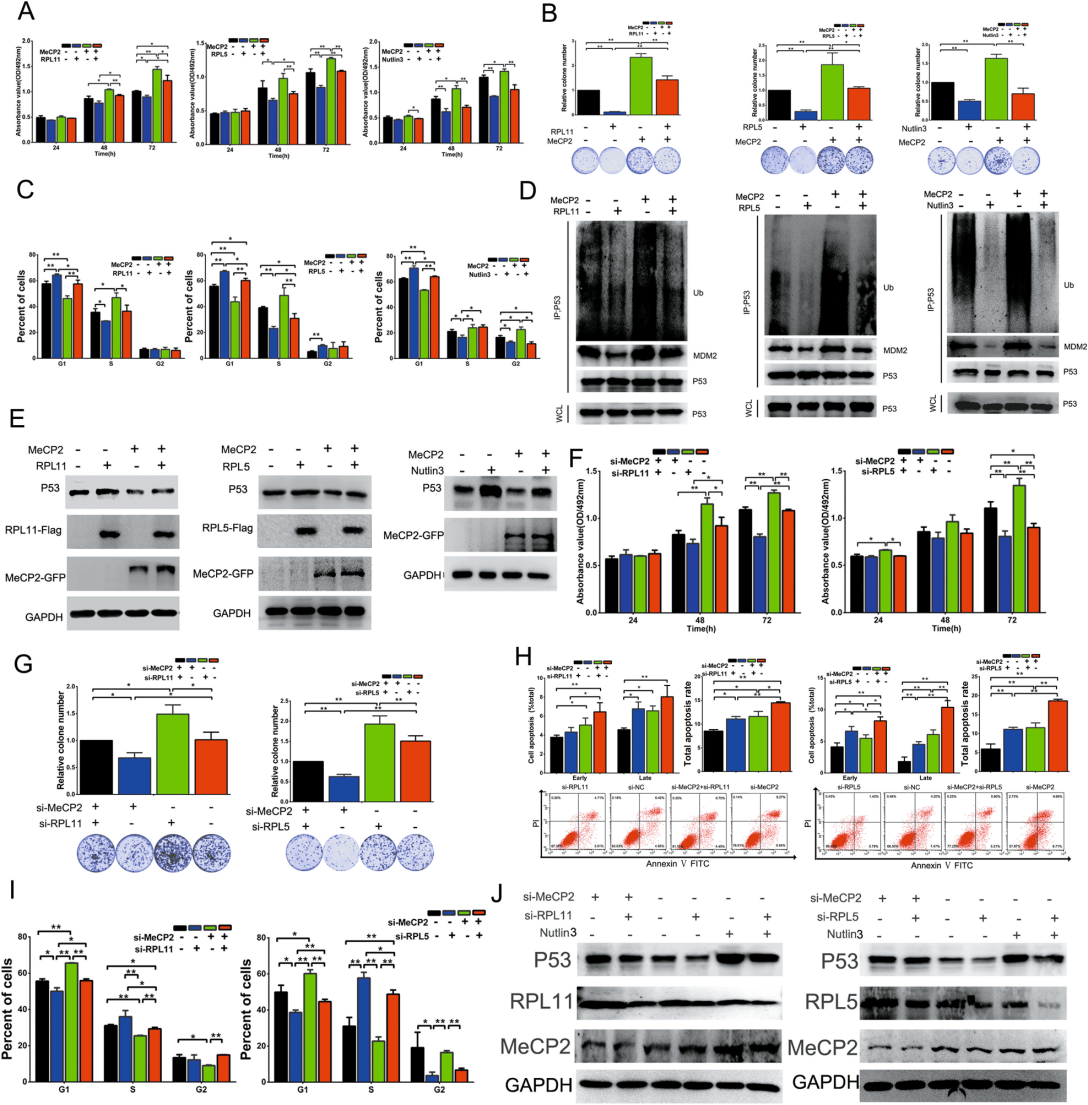
MeCP2 promotes breast cancer cell proliferation through regulating P53 degradation by inhibiting the expression of RPL11 or RPL5. **a** MTT assay. *P < 0.05, **P < 0.01. **b** Cell colonies assay. *P < 0.05, **P < 0.01. **c** Cell cycle analysis. *P < 0.05, **P < 0.01. **d** Ubiquitination assay. **e** The P53 protein levels in MCF7 cells co-transfected with MeCP2 and RPL11 or MeCP2 and RPL5, and in cells transfected with MeCP2 overexpression vector and treated with MDM2 inhibitor (Nutlin3). **f** MTT assay. *P < 0.05, **P < 0.01. **g** Cell colonies. *P < 0.05, **P < 0.01. **h** Cell apoptosis assay. *P < 0.05, **P < 0.01. **i** Cell cycle analysis in MCF7 cells co-transfected with MeCP2 siRNA and RPL11/ RPL5 siRNA. *P < 0.05, **P < 0.01. **j** P53 protein expressions in MCF7 cells transfected with MeCP2 siRNA, RPL5 siRNA, or RPL11 siRNA, or treated with Nutlin3.

To further confirm that MeCP2 might promote breast cancer cell proliferation by suppressing RPL11/RPL5 expression and promoting the E3 ubiquitin ligase activity of MDM2, MeCP2 overexpression vector was co-transfected with RPL11 or RPL5 overexpression vectors or MDM2 inhibitor (Nutlin3) into MCF7 cells. Cell viability and colony formation assays showed that upregulation of MeCP2 expression promoted cell proliferation, while overexpression of RPL11/RPL5 or Nutlin3 partially reversed this effect (Fig. 5a, b). G1-phase cells decreased significantly with MeCP2 overexpression and were restored by co-transfection of MeCP2 overexpression vector with RPL11/RPL5 overexpression vectors or Nutlin3 (Fig. 5c and Supplementary Fig. S4A–D). Furthermore, MeCP2 overexpression induced P53 ubiquitination and downregulated P53 protein expression, while overexpression of RPL11/RPL5 or Nutlin3 partially reversed these effects (Fig. 5d, e).

Next, we synthesized RPL11 and RPL5 siRNAs, which were co-transfected with MeCP2 siRNA into MCF7 cells. Cell viability and colony formation assays showed that downregulation of MeCP2 expression led to suppressed cell proliferation, which was rescued by silencing RPL11 or RPL5 (Fig. 5f, g). The apoptosis level was dramatically lower in cells co-transfected with MeCP2 siRNA and RPL11/RPL5 siRNAs than in those with MeCP2 siRNA alone (Fig. 5h). Cell cycle assay also showed that silencing MeCP2 induced a significant increase in G1-phase cells, while co-transfection with MeCP2 siRNA and RPL11/RPL5 siRNAs reversed this effect (Fig. 5i). Moreover, MeCP2 siRNA or Nutlin3 upregulated P53 protein expression, which was abrogated by RPL11 or RPL5 siRNAs (Fig. 5j). Altogether, these results confirmed that MeCP2 promoted breast cancer cell proliferation and inhibited cell apoptosis through promoting ubiquitination-mediated P53 degradation by suppressing RPL11 and RPL5 expression.

### MeCP2 promoted breast cancer cell proliferation by regulating the RPL11/RPL5-P53 pathway in vivo

**Fig. 6:**
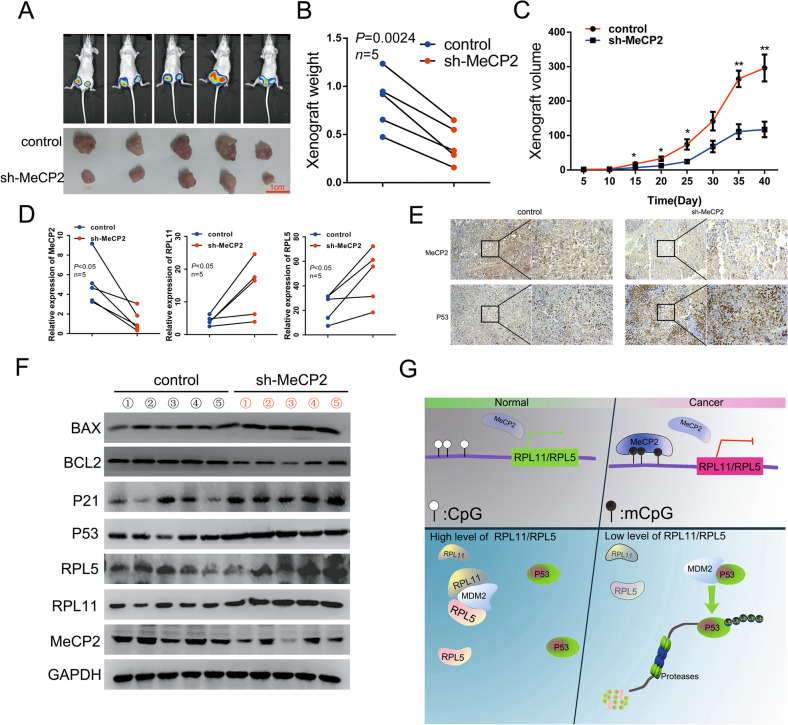
MeCP2 facilitates breast cancer growth by regulating RPL11/RPL5-P53 pathway in vivo. **a** Small animal imaging of tumor-bearing mice (up) and gross morphology of xenograft (bottom) 40 days after injection. **b** Tumor weight at day 40 after injection. **c** Growth curves of tumor. Tumor volume was generated every 5 days for 40 days. *P < 0.05, **P < 0.01. **d** qRT-PCR of MeCP2, RPL11 and RPL5 expressions in tumor xenografts. **e** IHC staining of MeCP2 and P53 in xenografts. **f** The protein levels of Bcl2, P53, BAX, P21, RPL11, and RPL5 in vivo. **g** Proposed model for the effect of MeCP2 on breast cancer growth. MeCP2 facilitated breast cancer cell proliferation by binding to RPL11 or RPL5 promoter region and suppressing their expressions, thereby promoting ubiquitination-mediated P53 degradation.

To further investigate the role of MeCP2 in breast cancer cell proliferation in vivo, we constructed an artificial shRNA lentiviral vector containing a selected MeCP2-targeting sequence (sh-MeCP2) and generated a stable MCF7 cell clone. Control-infected and sh-MeCP2-infected MCF7 cells were injected subcutaneously into both groin flanks of nude mice, and tumor growth was observed. It was found that the tumor growth, based on tumor weight and size, was significantly inhibited by sh-MeCP2 (Fig. 6a–c). MeCP2 mRNA expression was markedly downregulated in the sh-MeCP2 group compared with that in the control group, while the mRNA expression levels of RPL11 and RPL5 were upregulated (Fig. 6d). In addition, the protein expression of MeCP2 decreased in the sh-MeCP2 xenografts while that of P53 increased (Fig. 6e, f and Supplementary Fig. S5). Furthermore, Bcl2 expression was downregulated in the sh-MeCP2 xenografts, whereas the levels of BAX, P21, RPL11, and RPL5 were upregulated (Fig. 6f). These data further verified that MeCP2 promoted breast cancer cell proliferation via regulating P53 degradation by inhibiting RPL11 and RPL5 expression in vivo.

## Discussion

Accumulating evidence has indicated that MeCP2, as an important epigenetic regulator, may be a key oncogene in various cancer types^11,12^. Reportedly, MeCP2 promotes colorectal cancer cell growth, regulates the carcinogenesis and growth of osteosarcoma and neuroblastoma, and facilitates oral squamous cell carcinoma proliferation^19,20^. Silencing MeCP2 inhibits cell proliferation in transformed human prostate cells^21^. Our previous study demonstrated dramatically upregulated MeCP2 expression in gastric cancer, which facilitates cancer cell proliferation and suppresses cell apoptosis in vitro and in vivo^16,17^. The present study aimed to identify the function and molecular mechanism of MeCP2 in human breast cancer. Our results revealed strong MeCP2 upregulation in primary breast cancer and indicated that a high MeCP2 level was closely associated with cancer M stage and overall survival, therefore suggesting that MeCP2 may play a crucial role in the carcinogenesis and proliferation of breast cancer.

Our results demonstrated that silencing MeCP2 significantly inhibited breast cancer cell proliferation and impeded tumor growth as well as repressed cell growth by blocking G1–S cell cycle transition through regulating CDK2, P53, and P21^22–27^, which are cell cycle-regulatory genes involved in tumor carcinogenesis and proliferation^28,29^. In addition, silencing MeCP2 remarkably suppressed breast cancer cell migration by inhibiting β-catenin expression and induced cell apoptosis by downregulating the antiapoptotic gene Bcl-2 and upregulating proapoptotic genes, including Bax, P53, and P21. The Bcl-2 family, an apoptosis-related gene family, may be divided into antiapoptotic and proapoptotic genes. Bcl-2 can inhibit apoptosis through forming heterodimers with Bax after inactivating it^30^. The balance between Bcl-2 and Bax is considered crucial. Amplified Bax accelerates apoptosis, whereas excessive Bcl-2 suppresses it. P53 and P21 exert their tumor-suppressive functions by regulating cell-cycle checkpoints and apoptosis^31,32^. Taken together, our study confirms the mechanism that MeCP2 promotes breast cancer cell proliferation and inhibits apoptosis by repressing the P53 signaling pathway.

Our GSEA and ChIP-Seq results suggest that RPL11 and RPL5 are MeCP2-targeting genes. It was demonstrated that MeCP2 binds to the methylated CpG islands in the promoter regions of both genes, resulting in downregulated expression. RPL11 and RPL5 are members of the ribosomal protein (RP) family. RPL11 is involved in gastric cancer, colorectal cancer, fibroblasts, lymphoma, and esophageal squamous carcinoma, where it acts as a cancer suppressor gene^33–37^. RPL5 also functions as a tumor suppressor in multiple cancer types, including multiple myeloma, lymphoblastic leukemia, breast cancer, papillary thyroid carcinoma, and rhabdoid tumors^38,39^. RPL11 and RPL5 may inhibit cancer cell proliferation and induce apoptosis^40^. In this study, TCGA data revealed that RPL11 and RPL5 expression was significantly lower in breast cancer tissues, and their expression levels were associated with overall survival. Our results indicated that overexpression of RPL11 or RPL5 suppressed breast cancer cell proliferation, blocked G1–S cell-cycle transition, and induced cancer cell apoptosis. Our rescue experiments demonstrated for the first time that MeCP2 promoted breast cancer cell growth and induced apoptosis through suppressing RPL11 and RPL5 transcription by binding to the methylated CpG islands of their promoter regions.

The perturbation of various steps in the complex proteosynthetic process is commonly considered as nucleolar or ribosome biogenesis stress^41–44^, resulting in cell cycle arrest and senescence or apoptosis through activation of the P53 tumor suppressor protein. MDM2 is an E3 ubiquitin ligase that targets P53 protein for proteasomal degradation^45,46^. Therefore, MDM2 suppression leads to the stabilization and accumulation of P53. The MDM2-P53 pathway is regulated by certain RPs^47,48^. In response to nucleolar stress, RPL4, RPL5, RPL11, RPL23, RPS7, and RPS27 translocate from the nucleolus to the nucleoplasm and bind to MDM2, inhibiting its ubiquitin ligase activity toward p53, which leads to p53 accumulation^49–52^. In this study, we observed that RPL11 and RPL5 suppressed ubiquitination-mediated P53 degradation by directly binding to MDM2. Our experimental results suggested that MeCP2 promoted breast cancer cell proliferation and inhibited apoptosis through promoting ubiquitination-mediated P53 degradation by suppressing RPL11 and RPL5 expression.

In summary, the present study demonstrates that MeCP2 is an oncogene that is highly expressed in breast cancer. MeCP2 promotes breast cancer cell proliferation and cell cycle progression and inhibits cell apoptosis via suppressing the transcription of RPL11 and RPL5 by binding to their promoter regions. Lower protein expression of RPL11 and RPL5 leads to fewer RPL11/ MDM2 and RPL5/MDM2 complexes, and increased free-state MDM2 promotes ubiquitination-mediated P53 degradation. Our findings suggest that MeCP2 plays an important role in breast cancer proliferation and may represent a promising therapeutic target for the disease.

## Materials and methods

### Cell lines

Human breast cancer cell lines MCF-7 and ZR-75-1 were obtained from Genechem (Shanghai Genechem Co., Ltd., Shanghai, China). Both cell lines were identified by short tandem repeat analysis. MCF-7 cells were maintained in 1640 medium (1640; PAA Laboratories GmbH, Cölbe, Germany) supplemented with 10% FBS (Biological Industries, Cromwell, CT, USA), and ZR-75-1 cells were maintained in RPMI 1640 medium (PAA Laboratories GmbH) supplemented with 15% FBS (Biological Industries) and 1% Insulin, Transferrin, Selenium Solution (ITS-G; Gibco, Grand Island, NY, USA). The cells were cultured in a humidified 5% CO_2_ incubator at 37 °C. To establish a stable MeCP2 knockdown cell line, MCF-7 cells were firstly transfected by Sh-MeCP2 or control lentivirus. After geneticin resistance screening, the two cell lines were additionally transfected by the luciferase reporter gene lentivirus to facilitate in vivo imaging experiments.

### Animals

Four-week-old female BALB/c nude mice were provided by the Laboratory Animal Center, Xi’an Jiaotong University Health Science Center. The mice were fed under pathogen-free conditions. All experimental procedures followed the criteria of the Institutional Animal Care and Use Committee of the university. The cells transfected with 2×106 Sh-MeCP2 or control lentivirus were resuspended in 100 μl PBS and injected into the contralateral inguen of each mouse. Xenografts were measured 5 days after injection and every 4 days thereafter. The xenograft volume was calculated as *V* = *L* × (*W* × ½)2 (*L*: longest dimension; *W*: shortest dimension). After 40 days, the luciferase activity of xenografts was detected by small animal imaging. Then, mice were euthanized under deep anesthesia, the xenografts were surgically removed, and the incision was sutured. The xenografts were preserved for use in subsequent experiments.

### Cell transfection

PEGFP-C1 vector containing MeCP2 cDNA was purchased from GENEWIZ (Suzhou, China). MBD deletion gene (MeCP2 ΔMBD), TRD deletion gene (MeCP2ΔTRD), and TRD partial deletion gene (MeCP2ΔTRD + NLS) were amplified twice using overlap PCR (the primers are shown in Table S3). RPL11, RPL5, and the corresponding control vectors were purchased from Genechem (Shanghai, China). The following siRNAs were purchased from GenePharma (Shanghai, China): control siRNA, forward (5′–3′): UUCUCCGAACGUGUCACGUTT, reverse (5′–3′): ACGUGACACGUUCGGAGAATT; MeCP2 siRNA#1, forward: GCUUCCCGAUUAACUGAAATT, reverse: UUUCAGUUAAUCGGGAAGCTT; MeCP2 siRNA#2, forward: GCUUAAGCAAAGGAAAUCUTT, reverse: AGAUUUCCUUUGCUUAAGCTT; RPL11 siRNA, forward: GGUGCGGGAGUAUGAGUUATT, reverse: UAACUCAUACUCCCGCACCTT; RPL5 siRNA, forward: GGGAGCUGUGGAUGGAGGCTT, reverse: GCCTCCATCCACAGCTCCCTT. Sh-MeCP2 and control lentiviruses were bought from Genechem and used following the standard protocol. Vector or siRNA transfection was performed according to standard protocols by using PolyPlus (PolyPlus, Illkirch-Graffenstaden, France).

### Public clinical datasets analysis

Data regarding MeCP2, RPL11, and RPL5 expression and clinical information were downloaded from Broad Institute’s Firehose (https://xenabrowser.net)^53^. For GSEA, breast cancer patients were classified into high- and low-MECP2 expression groups according to the median MeCP2 expression. The analysis was done in the desktop GSEA software^54,55^ using 1000 phenotype permutations, and gene sets with a nominal P value < 0.05 and false discovery rate FDR < 0.25 were considered significant. TCGA breast cancer data were evaluated using the pan-cancer data of the online databases Kaplan Meier Plotter^56^ and GEPIA^57^, and the best cutoff values were selected according to the defined high or low expression of related genes.

### Cell viability assay

MCF7 and ZR-75-1 cells were counted and seeded into 96-well plates at 1000 cells per well. After adherence, breast cancer cells were transfected with vectors or siRNAs. After 24, 48, and 72 h of transfection, cells were incubated with 200 μl culture medium containing 10 μl MTT (5 mg/ml) in 37 °C for 4 h in a humidified 5% CO_2_ atmosphere. The supernate was pipetted out carefully, and the bottom substrate was treated with 150 μl DMSO per well and incubated for 15 min in the dark at 25 °C. Absorbance was detected at 492 nm using a microplate reader.

### Clone formation assay

MCF7 and ZR-75-1 cells were seeded in 6-well plates and transfected with vectors or siRNAs. After 24 h, the cells were harvested, diluted, seeded into new 6-well plates at low density (1000 cells per well), and cultured for 14 days. The colonies were fixed in 4% paraformaldehyde and stained with 1% crystal violet. The clones were photographed and counted in an image acquisition system.

### Wound-healing assay

MCF7 cells were cultured in 6-well plates and transfected after reaching 50% cell density. After transfection, the cells were wounded using a 10 μl pipette tip and then washed three times with PBS. The cells were cultured in 1640 medium (1640; PAA Laboratories GmbH) supplemented with 1% FBS (Biological Industries) at 37 °C in a humidified 5% CO_2_ atmosphere. The wounds were photographed at 0, 24, 48, and 72 h after transfection.

### Invasion assay

For invasion assay, MCF7 cells were cultured in 6-well plates, transfected with siRNAs, harvested, and counted. Then, 6000 cells were diluted in 200 μl FBS free medium and seeded into the upper Transwell chamber (MCMP24H48, Merck KGaA, Darmstadt, Germany), a 24-well plate was placed in the Transwell chamber, and the bottom wells were filled with 500 μl 15% FBS medium. After 24 h of culture, cells were carefully removed, and the cells that invaded through the membrane were fixed in 4% paraformaldehyde and stained with 1% crystal violet staining solution. The invasive cells were photographed and counted.

### Flow cytometry assay

Cell cycle and apoptosis were analyzed as previously described16. Cell phase was examined by flow cytometry (FACS Calibur, BD Biosciences, San Jose, CA, USA).

### Chromatin immunoprecipitation (ChIP)

ChIP was performed as previously described16. Briefly, cells were cross-linked in RPMI 1640 containing 1% formaldehyde for 15 min at room temperature. Chromatin was fragmented by ultrasound, and the lysate was immunoprecipitated with Dynal magnetic beads (Invitrogen, Carlsbad, CA, USA) and antibodies for MeCP2 (ab2828, Abcam, Cambridge, MA, USA) or GFP (ab290, Abcam). After DNA elution and purification, qPCR and/or PCR were performed. IgG was used as a negative control.

### Immunofluorescence

Cells were seeded into Nunc Glass Bottom Dishes (Thermo Scientific, Waltham, MA, USA). After 48 h, the cells were fixed in 4% paraformaldehyde, permeabilized with PBST, and blocked in PBSB. Then, the cells were incubated with the primary antibodies RPL11 (#18163, CST, Danvers, MA, USA), RPL5 (#51345, CST), P53 (10442-1-AP, Proteintech, Wuhan, China), and Flag (#8146, CST) overnight at 4 °C. After incubation with the secondary antibodies and DAPI, cells were observed under a fluorescence microscope.

### Co-immunoprecipitation

After harvesting cells using RIPA buffer, the lysate was incubated overnight with primary antibodies MDM2 (66511-1-Ig, Proteintech), P53 (10442-1-AP, Proteintech) and Flag (#8146, CST). The protein-antibody complex was incubated with Dynal magnetic beads for 2 h and then boiled with loading buffer for 10 min. The obtained supernatant was used for western blotting analysis. Antibodies from different species were used to eliminate the effects of light and heavy chains of primary antibodies on the analysis. The following antibodies were used in western blotting: P53 (60283-2-Ig, Proteintech), MDM2 (19058-1-AP, Proteintech), and ubiquitin (sc-8017, Santa Cruz).

### Statistical analysis

Gene expression levels and correlations between MeCP2 and RPL11 or RPL5 from TCGA were analyzed by R package ggstatsplot^58^. The correlation matrix analysis was conducted using R package corrplot^59^. Other data were analyzed using SPSS 24.0. Student’s two-tailed t-test was used for comparisons between two groups. Differences between samples are presented as the mean ± SD.

## Supplementary information

All primers in this study

supplement figure legend

Figure S1

Figure S2

Figure S3

Figure S4

Figure S5

Cell Line MCF7 [MCF-7] STR Profile Report

Cell Line ZR-75-1 STR Profile Report
