# Supplementary material for: MeCP2 facilitates breast cancer growth via promoting ubiquitination-mediated P53 degradation by inhibiting RPL5/RPL11 transcription
Source: Oncogenesis. 2020 Jun 1;9(5):56. doi: 10.1038/s41389-020-0239-7 (PMC7264296; doi:10.1038/s41389-020-0239-7)
Supplement: Supplementary file 1 — All primers in this study [file 41389_2020_239_MOESM1_ESM.pdf]

Table S1 qRT-PCR primers

symbol	Forward	Reward
MeCP2	GCCGAGAGCTATGGACAGCA	CCAACCTCAGACAGGTTTCCAG
RPL5	ATGGGCCAGAATGTTGCAGA	TTTGGGACGGTTCCACCTCT
RPL11	CCTGGACTTCTATGTGGTGCT	CGGGGTTGTTACCAGCTACTC
RPS15	CTCTTCTGAGGATCCGGCAAG	TGCATCAGCTGCTCGTAGG
RPS6	TGGGTGAAGAATGGAAGGGTTA	AAGACACCCTGCTTCATGGG
RPS11	TGGGCTTCAAGACACCCAAG	ATCTTGGTCACCACGCCAGA
RPL23A	TTCACAAGATGGCGCCGAAA	GGTCAAGCTTGTTTCTCCTGG

Table S2 ChIP-PCR primers

symbol	Forward	Reward
RPL11-1	TCATCACACAAGGACCACCAGCTCG	GCATTTGGCAAAGCCCTGCAGCGTC
RPL11-2	CTGATTCGGGAGCTTTCAAAAACTA	GCCGAGGGCCTTATGGGCCAGGAGG
RPL11-3	TAGCACTACCCACAAGGGAGAAGCC	GATAAAGGAAAGCAAATCCAGGTCC
RPL11-4	GTGGTTAGCCTGGGAAAAGAGCCCG	GCATTGCGGGCTCCATATTCCTCGG
RPL11-5	TCTCCATCATGGCGGTGAGTAGCTG	ACGATGGCAGGGAAAGTGACAGAAT
RPL11-6	CTGCGGGGGCTACTTCGCCCGCAGC	CCCCATCTGCCCCCGCTGCAGCCTC
RPL11-7	GTTCGAGCCAAGGACGCAGGGTTGA	CTAGCACCGAGCAAGTCTCAAGTCC
RPL11-8	CCGGAGTGCTCAGAAGCACGGTCAG	CGACGTCCAGCATCCTATTCGGAAG
RPL11-9	GGTCCGGGCCTTTTCCTGGTCCCGG	TTGTAAGGCCAACTAAGGGGAGTGA
RPL11-10	AGGGAAGGTGCCAGCCTTTAGACAG	CCTCCACGAACGGAACGGGTGAAGA
RPL5-1	CGGAACGTAAAACGCCTTGTGGCCG	CCCGAGACCGAGGCATCCACTCACC
RPL5-2	TGCGCCTGCGCAAGGGCTGTGGCCC	CGGCCCCTGCGACGCGCGAGACGGG
RPL5-3	GACGCCGGTCTCTGTTCCGCAGGAT	ACCGGGCGGCAGCCATTTAAGGCCA
RPL5-4	TTCTTGCCCGTATGCCAGCCTAGGG	ACTCACCCTTCTCCCCAATCCGCCG
RPL5-5	CTTGGTCGAGGTGCAGTTCCCAGCG	GACCTCTCACGCCCGGCCGCTTGCC
RPL5-6	CGAAGAAGGGTTGCGTGAGCTTGGA	GTCGAGTGGGACCCGAGGACCTGCA
RPL5-7	CTGCCGTCTAGTGTGAGGGGGCGCT	ACAAGCTTCCTAAATCTAAGACTTG
RPL5-8	GGGGAACGCCAGAATGGAGGGGGGC	GGGGCCCAGGGACCCCTCGGCCTCC
RPL5-9	TTTTCCAGGAGAATGTCGGAGCCGG	CCCACCCTTGGGGCCCTACCCCAA

Table S3 Overlap PCR primers

symbol	sequence
adapter primer-F	CAAGCTTCGATGGCTGCCGCTGCTGCCGCTGCTCC
adapter primer-R	CGGGATCCTCAGCTCACTCTCTCGGTGACAGGG
ΔMBD overhang primer-F	TCTGTTCCCTCCTGCTAGGGCTATCCCTGATGATGGACCTCCTT
ΔMBD overhang primer-R	AAGGAGGTCCATCATCAGGGATAGCCCTAGCAGGAGGGAACAGA
ΔTRD overhang primer-F	TCCTTGACTTCGATGCTGACGGTGCCCTCGCTGGTAGCGGCTTTG
ΔTRD overhang primer-R	CAAAGCCGCTACCAGCGAGGGCACCGTCAGCATCGAAGTCAAGGA
ΔTRD+NLS overhang primer-F	TAGGAATGGCCTGGGGGTCGGCCTCAGCCTTCCTGCCCTCGCTGGTAGCGGCT
ΔTRD+NLS overhang primer-R	CCCCCAGGCCATTCCTAAAAAGAGGGGCAGGAAGACCGTCAGCATCGAAGTCA
