# Supplementary material for: MeCP2 facilitates breast cancer growth via promoting ubiquitination-mediated P53 degradation by inhibiting RPL5/RPL11 transcription
Source: Oncogenesis. 2020 Jun 1;9(5):56. doi: 10.1038/s41389-020-0239-7 (PMC7264296; doi:10.1038/s41389-020-0239-7)
Supplement: Supplementary file 2 — supplement figure legend [file 41389_2020_239_MOESM2_ESM.docx]

Figure S1. PCA analysis of genes related to MeCP2 in breast cancer based on TCGA data. (A) 3D scatter plot and variance distribution based on expressions of genes involved in the cancer related pathway from GSEA analysis. (B) 2D scatter plot.

Figure S2. The expression of MeCP2 mRNA in breast cancer cells after transfection with MeCP2 siRNAs. (A) qRT-PCR of MeCP2 mRNA expression in MCF7 cells. ***P*<0.01. (B) qRT-PCR of MeCP2 mRNA expression in ZR-75-1 cells. **, *P*<0.01.

Figure S3. Effect of MeCP2 on the exprefrssions of ribosomal proteins. (A) PCA analysis of genes involved in the ribosome pathway related to MeCP2 as identified by GSEA. (B) PCA analysis of genes involved in the ubiquitin pathway related to MeCP2 as identified by GSEA. (C) Kaplan-Meier analysis of the effect of MeCP2 to RP ratio on the overall survival of breast cancer based on TCGA data. (D) MeCP2 wild type and MeCP2 mutation vectors structure diagram. (E) The domain responsible for binding to RPL11/RPL5 promoter. Different mutations of MeCP2 vector were transfected in MCF7 cells. The interaction was verified by ChIP PCR gel electrophoresis analysis. (F) Correlation between the methylation levels of the specific CpG sites in RPL11/RPL5 promoter and the RPL11/RPL5 expression, respectively.

Figure S4. The raw charts of flow cytometry. (A) Cell cycle in ZR-75-1 cells after co-transfection with MeCP2 and RPL11 overexpression vectors. (B) Cell cycle after co-transfection with MeCP2 and RPL5 overexpression vectors. (C) Cell cycle after co-transfection with MeCP2 siRNA and RPL11 siRNA. (D) Cell cycle after co-transfection with MeCP2 siRNA and RPL5 siRNA.

Figure S5. IHC staining of MeCP2 and P53 in tumor tissues from mice injected with Control or sh-MeCP2 (n=5).
