# Supplementary figures and images for: MeCP2 facilitates breast cancer growth via promoting ubiquitination-mediated P53 degradation by inhibiting RPL5/RPL11 transcription
Source: Oncogenesis. 2020 Jun 1;9(5):56. doi: 10.1038/s41389-020-0239-7 (PMC7264296; doi:10.1038/s41389-020-0239-7)

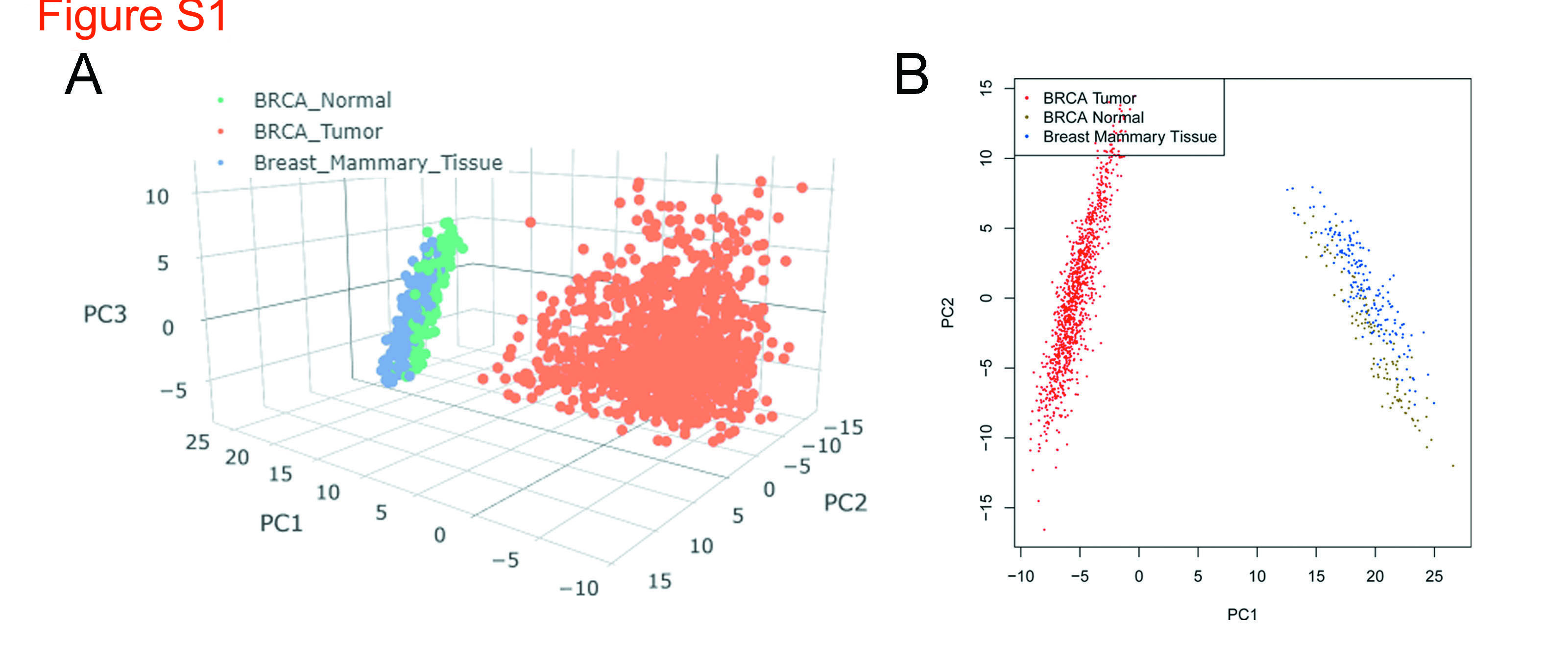

Supplement: Supplementary file 3 — Figure S1 [file 41389_2020_239_MOESM3_ESM.jpg]

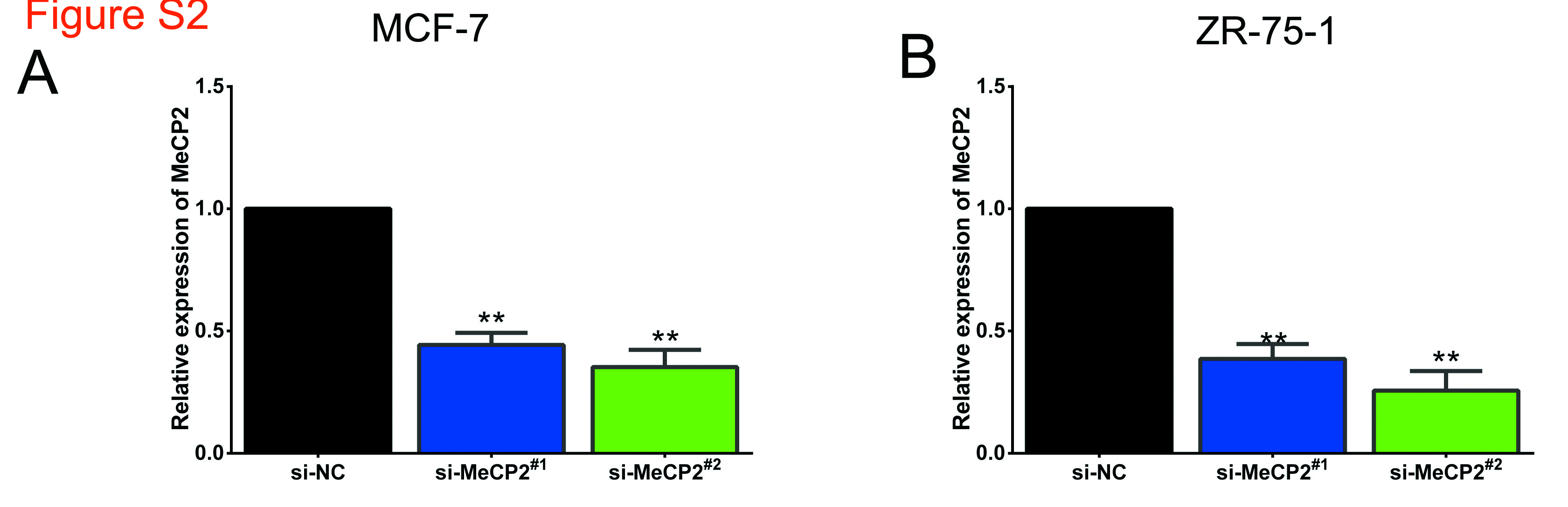

Supplement: Supplementary file 4 — Figure S2 [file 41389_2020_239_MOESM4_ESM.jpg]

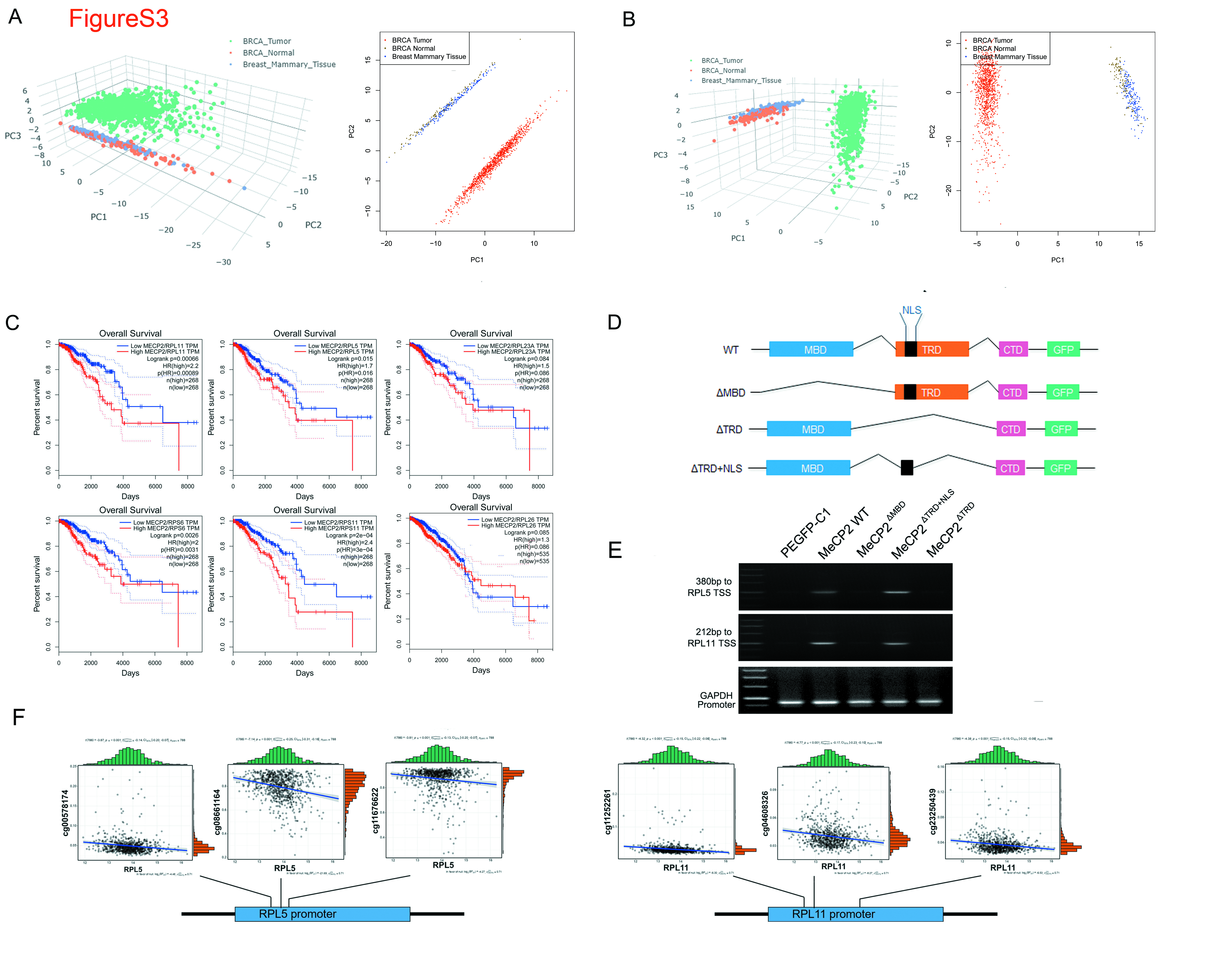

Supplement: Supplementary file 5 — Figure S3 [file 41389_2020_239_MOESM5_ESM.jpg]

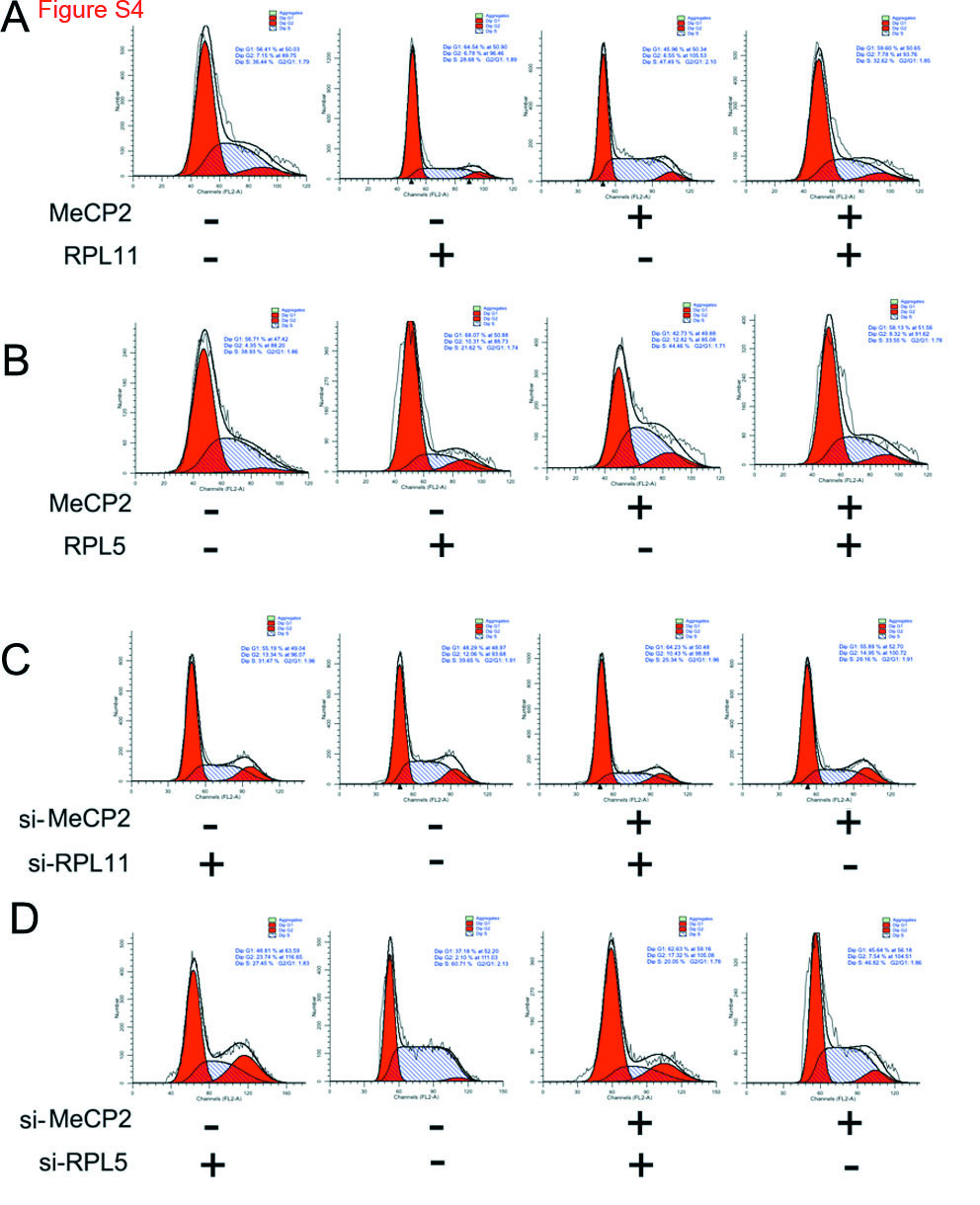

Supplement: Supplementary file 6 — Figure S4 [file 41389_2020_239_MOESM6_ESM.jpg]

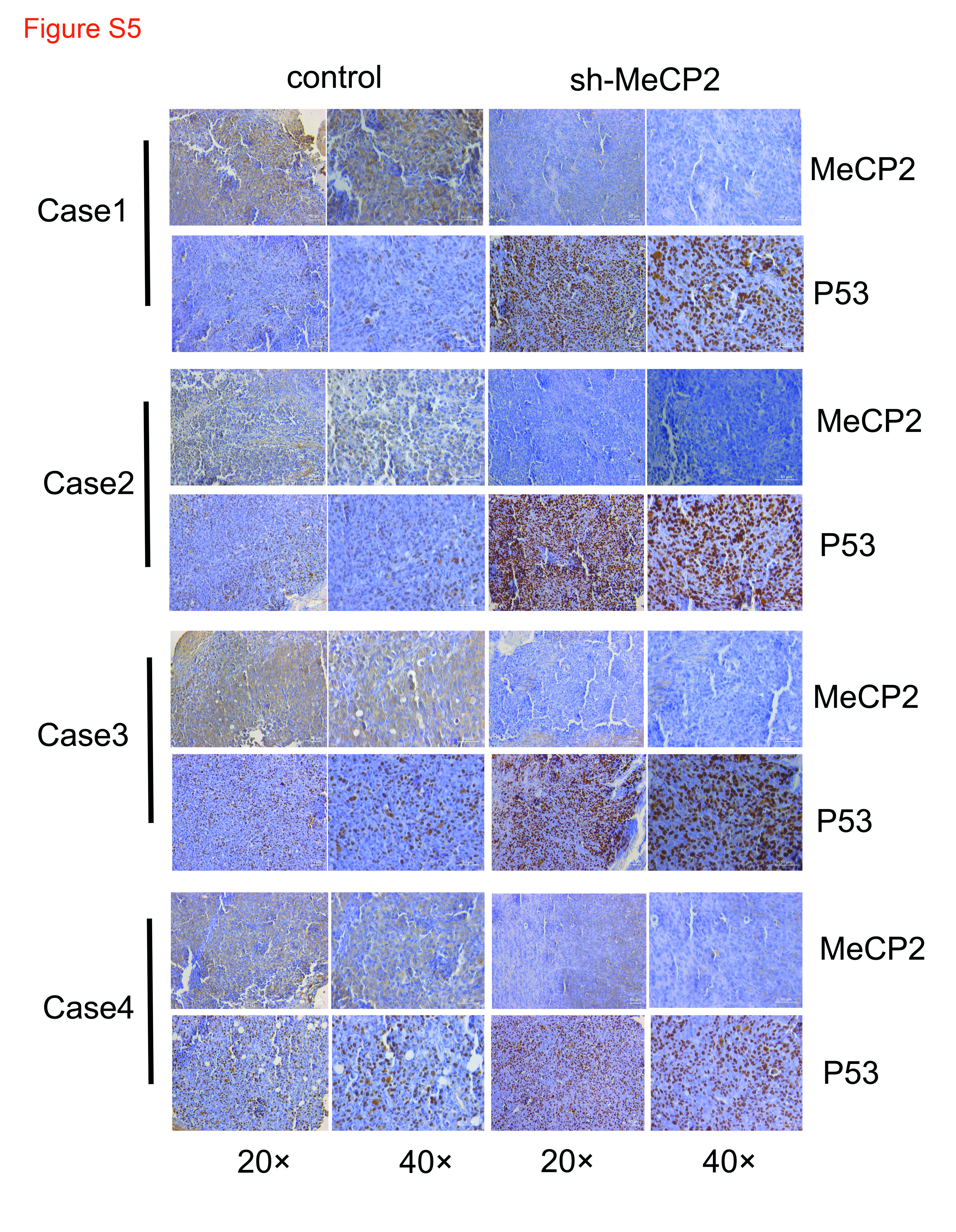

Supplement: Supplementary file 7 — Figure S5 [file 41389_2020_239_MOESM7_ESM.jpg]
