# Supplementary material for: MeCP2 facilitates breast cancer growth via promoting ubiquitination-mediated P53 degradation by inhibiting RPL5/RPL11 transcription
Source: Oncogenesis. 2020 Jun 1;9(5):56. doi: 10.1038/s41389-020-0239-7 (PMC7264296; doi:10.1038/s41389-020-0239-7)
Supplement: Supplementary file 8 — Cell Line MCF7 [MCF-7] STR Profile Report [file 41389_2020_239_MOESM8_ESM.pdf]

# Cell Line MCF7 [MCF-7] STR Profile Report

**Cell Line Designation:** MCF7 [MCF-7]

**Date Sample Received:** Oct 20th, 2017

**Report Date:** Oct 31th, 2017

**Methodology:** Twenty short tandem repeat (STR) loci plus the gender determining locus,Amelogenin, were amplified using the commercially available PowerPlex® 21 System from Promega Corporation. The amplified products were processed using the Applied Biosystems 3730xl DNA Analyzer. Data were analyzed using GeneMapper 5.0 software (Applied Biosystems). Appropriate positive and negative controls were run and confirmed for each sample submitted.

**Data Interpretation:** Cell lines were authenticated using Short Tandem Repeat (STR) analysis as described in 2012 in ANSI Standard (ASN-0002) by the ATCC Standards Development Organization (SDO) and in Capes-Davis et al., Match criteria for human cell line authentication: Where do we draw the line? Int J Cancer.2013;132(11):2510-9.

Genechem

**GENECHEM performs STR Profiling following ISO 9001:2008 and ISO/IEC 17025:2005 quality standards.**

There are no warranties with respect to the services or results supplied, express or implied, including, without limitation, any implied warranty of merchantability or fitness for a particular purpose. GENECHEM is not liable for any damages or injuries resulting from receipt and/or improper, inappropriate, negligent or other wrongful use of the test results supplied, and/or from misidentification, misrepresentation, or lack of accuracy of those results. Your exclusive remedy against GENECHEM and those supplying materials used in the services for any losses or damage of any kind whatsoever, whether in contract, tort, or otherwise, shall be, at GENECHEM 's option, refund of the fee paid for such service or repeat of the service.

GENE 吉凯基因

## TEST RESULTS:

| Test Results for Submitted Sample | | Reference Database Profile |
|---|---|---|
| **Loci** | **Query Profile: MCF7 [MCF-7]** | **Database Profile: MCF7 [MCF-7]** |
| **Amelogenin** | X | X |
| **D13S317** | 11 | 11 |
| **D16S539** | 11 12 | 11 12 |
| **CSF1PO** | 10 | 10 |
| **TH01** | 6 | 6 |
| **vWA** | 14 15 | 14 15 |
| **D7S820** | 8 9 | 8 9 |
| **D5S818** | 11 12 | 11 12 |
| **TPOX** | 9 12 | 9 12 |
| **D3S1358** | 16 | |
| **D1S1656** | 11 15.3 | |
| **D6S1043** | 12 18 | |
| **Penta E** | 7 12 | |
| **D18S51** | 14 | |
| **D2S1338** | 21 23 | |
| **Penta D** | 12 | |
| **D8S1179** | 10 14 | |
| **D12S391** | 18 20 | |
| **D19S433** | 13 14 | |
| **FGA** | 23 24 25 | |
| **D21S11** | 30 | |

*Note: The top nine Loci (8 core STR loci plus Amelogenin) can be made public to verify cell identity. In order to protect the identity of the donor,* ***please do not publish*** *the allele calls from all the STR loci tested.*

Genechem

Percent Match: **100%**

The submitted profile is an exact match for the following human cell line(s) in the reference database (8 core loci plus Amelogenin): **MCF7 [MCF-7]**

### Explanation of Test Results

Cell lines with ≥80% match are considered to be related; i.e., derived from a common ancestry. Cell lines with between a 55% to 80% match require further profiling for authentication of relatedness. Reference Database Profile was obtained from ExPASy, DSMZ, ATCC or China National Infrastructure of Cell Line Resource STR database.

Applied Biosystems
GeneMapper Software 5

0810-final

| Sample File | Sample Name | Panel | OS | SQ |
|---|---|---|---|---|
| 90_A04.fsa | A04 | PowerPlex_21_POP7_Panels_v2.0 | | |

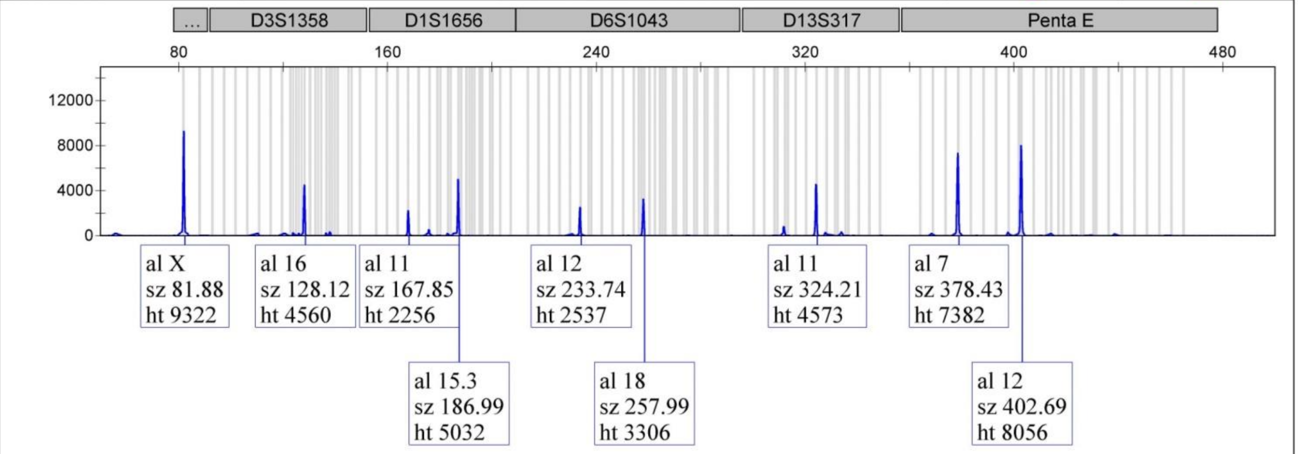


| Sample File | Sample Name | Panel | OS | SQ |
|---|---|---|---|---|
| 90_A04.fsa | A04 | PowerPlex_21_POP7_Panels_v2.0 | | |

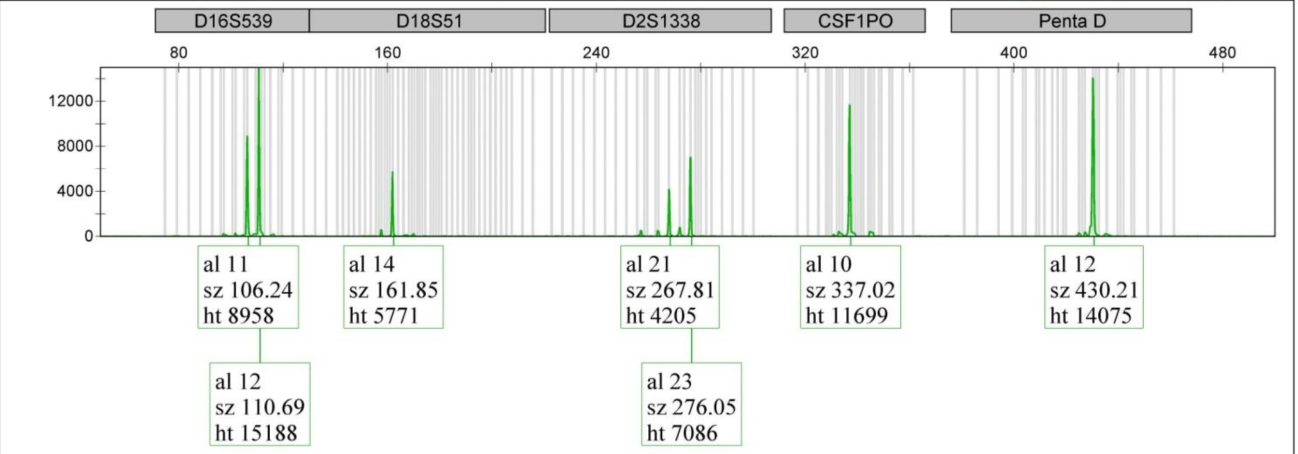


Wed Oct 25,2017 12:57PM, CST Printed by: gm Page 1 of 2

Applied Biosystems

GeneMapper Software 5

0810-final

| Sample File | Sample Name | Panel | OS | SQ |
|---|---|---|---|---|
| 90_A04.fsa | A04 | PowerPlex_21_POP7_Panels_v2.0 | | |

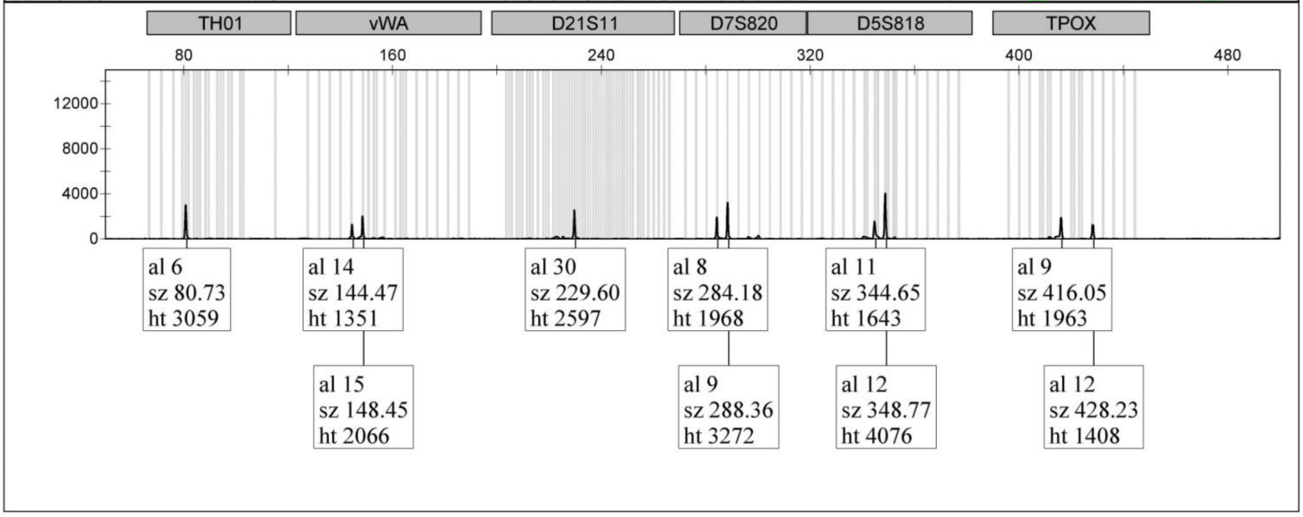


| Sample File | Sample Name | Panel | OS | SQ |
|---|---|---|---|---|
| 90_A04.fsa | A04 | PowerPlex_21_POP7_Panels_v2.0 | | |

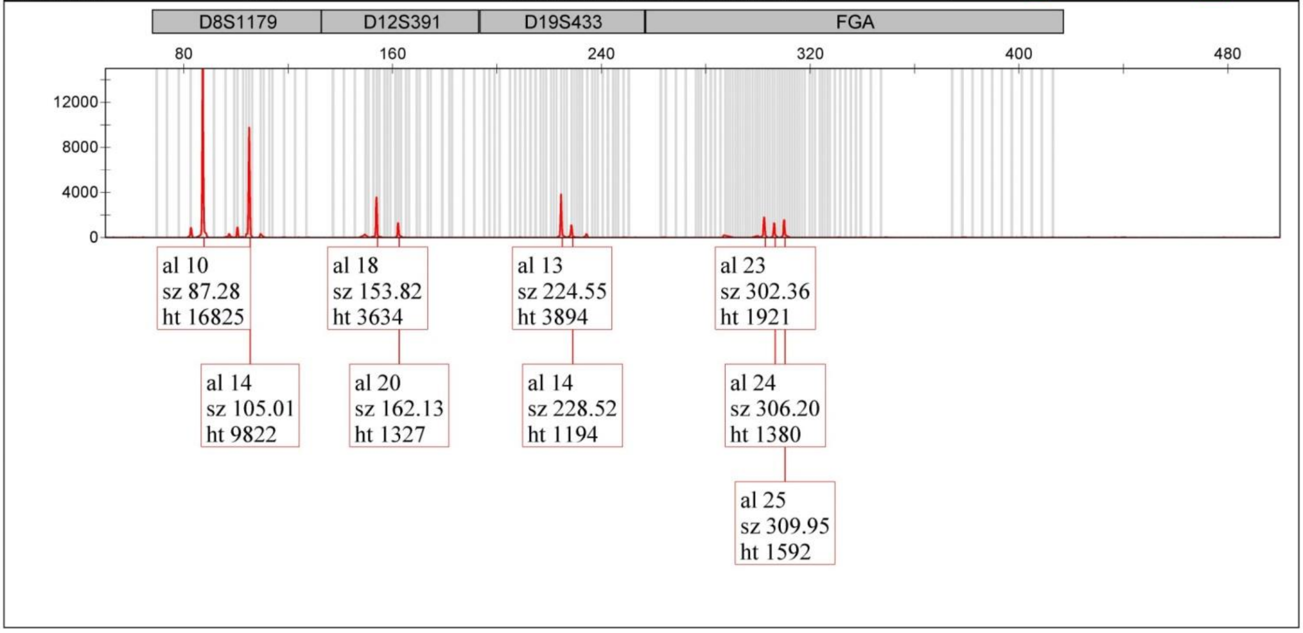


Wed Oct 25,2017 12:57PM, CST Printed by: gm Page 2 of 2


- Shanghai Genechem Co., Ltd.
- Add：NO.332, Aidisheng Road, Zhangjiang Hi-Tech Park, Shanghai 201203 P.R. China Tel : (86) 21 51320189 Fax : (86) 21 51370635
